# The Role of Regulatory T Cell in Nontypeable *Haemophilus influenzae*-Induced Acute Exacerbation of Chronic Obstructive Pulmonary Disease

**DOI:** 10.1155/2018/8387150

**Published:** 2018-03-13

**Authors:** Xuewa Guan, Yanjiao Lu, Guoqiang Wang, Peter Gibson, Fang Chen, Keyong Fang, Ziyan Wang, Zhiqiang Pang, Yingqiao Guo, Junying Lu, Yuze Yuan, Nan Ran, Fang Wang

**Affiliations:** ^1^Department of Pathogeny Biology, College of Basic Medical Sciences, Jilin University, Changchun 130021, China; ^2^Department of Respiratory and Sleep Medicine, John Hunter Hospital, Newcastle, NSW, Australia; ^3^Department of Intensive Care Unit, First Hospital of Jilin University, Changchun 130021, China

## Abstract

Chronic obstructive pulmonary disease (COPD) is associated with irreversible persistent airflow limitation and enhanced inflammation. The episodes of acute exacerbation (AECOPD) largely depend on the colonized pathogens such as nontypeable *Haemophilus influenzae* (NTHi), one of the most commonly isolated bacteria. Regulatory T cells (Tregs) are critical in controlling inflammatory immune responses and maintaining tolerance; however, their role in AECOPD is poorly understood. In this study, we hypothesized a regulatory role of Tregs, as NTHi participated in the progress of COPD. Immunological pathogenesis was investigated in a murine COPD model induced by cigarette smoke (CS). NTHi was administrated through intratracheal instillation for an acute exacerbation. Weight loss and lung function decline were observed in smoke-exposed mice. Mice in experimental groups exhibited serious inflammatory responses via histological and cytokine assessment. Expression levels of Tregs and Th17 cells with specific cytokines TGF-*β*1 and IL-17 were detected to assess the balance of pro-/anti-inflammatory influence partially. Our findings suggested an anti-inflammatory activity of Tregs in CS-induced model. But this activity was suppressed after NTHi administration. Collectively, these data suggested that NTHi might play a necessary role in downregulating Foxp3 to impair the function of Tregs, helping development into AECOPD.

## 1. Introduction

Chronic obstructive pulmonary disease (COPD) has been considered as the fourth leading cause of death globally. It is characterized by progressive, persistent airflow limitation, usually associated with enhanced chronic inflammatory responses in the airways and the lung according to the Global Initiative for Chronic Obstructive Lung Disease. Cigarette smoke (CS), as the main cause of COPD, influences pulmonary environment and increases susceptibility to respiratory infections [1]. During the periods of exacerbation with intensified symptoms, pathogen infection has been considered being a central component. Repeated exacerbations and recruited inflammatory mediators [2] are believed to distort function of innate and adaptive immune responses, which might lead to an altered respiratory host defense and progressive airflow limitation, contributing to the progress of COPD [3].

Nontypeable *Haemophilus influenzae* (NTHi) is believed to be one of the primary pathogens causing AECOPD as it spreads down to the lower respiratory tract [4]. Colonized NTHi in the airway could impair ciliary function [5], enhance mucus expression [6], and provide opportunities for further infection. As the most frequently isolated agent from COPD patients [7], NTHi might aggravate the inflammatory responses generated by smoking, leading to a progressive airway obstruction, declined lung function, and remodeled pulmonary tissue.

Regulatory T cells (Tregs), a subgroup of CD4^+^ T cells with a specific biomarker Foxp3, have been uncovered to play an important role in the development of COPD. In the airway of COPD, functions of Tregs exist as secreting anti-inflammatory factors and recruiting other anti-inflammatory cells [8, 9]. Involved in the differentiation and expansion of Tregs, TGF-*β* plays an immune-modulated role in maintaining immune homeostasis [10]. Elevated TGF-*β*1 levels found in COPD patients were prone to an environment for Tregs differentiation [11]. In contrast with the proinflammatory pathway via Th17 cells, the role of Tregs in this pro- and anti-inflammatory balance has been widely studied despite controversial results [12]. Barcelo et al. found an upregulation of Tregs in smokers' BALF compared with never-smokers [13]. While another clinical research [14] indicated higher frequencies of Th17 cells with related IL-17A and IL-22 in serum and lower expression of Foxp3 in both moderate and severe COPD patients.

Compelling researches have been keen on the correlation between Tregs and bacteria. Some pathogen-specific Tregs are known to prevent pathological injury induced by infection. However, increased infection and persistence of pathogens might also take place due to the suppressing function of Tregs. Studies have shown enhanced host susceptibility to *Listeria* and *Salmonella* species when Treg expression was excessive [15]. In a chronic otitis media model, the percentage of Tregs increased after NTHi inoculation, while a depletion of Tregs could induce a 99.9% reduction of bacterial counts, indicating an infectious tolerance of Tregs to NTHi [16]. However, a decrease of Tregs-associated Foxp3 gene expression was observed in animals colonized with NTHi on day of life 3, indicating a downregulated effect of NTHi on Tregs [17]. Following these researches, the aim of this study is to continuously investigate the correlation between NTHi and Tregs in COPD murine model.

COPD might be caused by cigarette smoke. A combined colonization of NTHi could aggravate it into AECOPD. Based on the presence of a pro-/anti-inflammatory balance reflected by Th17/Treg cell response in COPD, we hypothesized that NTHi infection could impair the anti-inflammatory Treg balance and lead to AECOPD eventually.

## 2. Materials and Methods

### 2.1. Bacteria Incubation

NTHi (ATCC 49247) was purchased from Xiang Biological Technology Co. Ltd. (Shanghai, China). Bacteria were incubated on a chocolate agar for 18–24 h at 37°C in 5% CO_2_. After a Gram staining identification, NTHi was continuously cultured for amplification and freeze preservation. Bacteria for administration was pelleted at 12,000 ×g for 10 min and washed twice in PBS. A diluted concentration of 1 × 10^8^ CFU/ml in PBS was required according to OD_600_ value.

### 2.2. Animals

Female 8-week BALB/c mice (Animal Center, Basic Medical Sciences College, Jilin University, SCXK(Ji)-2015-0001) were randomly divided into 4 groups, including a normal control group (NC group), a cigarette smoke group (CS group), a NTHi group, and a group treated with a combination of cigarette smoke and NTHi (NTHi + CS group). All animals were fed under the same conditions and weighed once a week. Mice in the NC group were exposed to room air. The CS and NTHi + CS group mice were exposed to CS for 16 consecutive weeks. On the first day of the 17th week, NTHi was administrated to cause infection in the NTHi and NTHi + CS groups. 24 hours later, mice were deeply anaesthetized with 1% pentobarbital sodium on the basic of weight for subsequent experiments.

### 2.3. COPD Murine Model

Given that cigarette smoke is the major cause of COPD [18], we utilized a passive smoking method as the foundation of COPD inflammation model in mice, as many studies did [19, 20]. Briefly, the CS and NTHi + CS group mice were placed in a whole-body exposure sealed plastic box (chamber dimensions: 45 cm × 31 cm × 16 cm, made in lab) with 8 ventholes (*d* = 5 mm) on top of the box for ventilation, 1 venthole on the side for connection to the smoke source (standardized 3R4F research cigarettes, University of Kentucky, Lexington, KY). An air pump was used for pumping smoke from lighted cigarettes into the box. Ventholes on top were blocked off while pumping. Two cigarette smokes were continuously inhaled into the sealed box each time to get a concentration of 300 mg/m^3^ total suspended particles, lasting for 10 min. Then, mice got a rest for 10 min with the cover removed. The above steps were carried out 6 times (smoking exposure time: 1 h) a day, 6 days per week, with a total duration of 16 weeks.

### 2.4. Treatment of NTHi

An intratracheal infection technique was used for administration of NTHi [21]. On the first day of the 17th week, mice in the NTHi and NTHi + CS groups were anaesthetized with pentobarbital sodium and vertically hung via their front teeth, in front of a cold light illuminator against the trachea. 50 *μ*l NTHi with a density of 1 × 10^8^ CFU/ml was administrated by dripping into the mouse trachea to cause infection in the NTHi and NTHi + CS groups. Same amount of PBS was administrated into the mice in other groups under the same conditions for control. 24 h later, mice were deeply anaesthetized for subsequent experiments.

### 2.5. Measurement of Animal Lung Function

Invasive lung function was investigated with anesthesia and tracheal instrumentation [22]. After being deeply anaesthetized with pentobarbital sodium, mice were fixed on panels for tracheal cannulation. The trachea was separated, and a cut was made in the upper trachea cartilaginous rings. A cannula was inserted into the trachea via the cut and fixed by operating line. Spirometer (Buxco, PFT Controller, DSI, USA) was utilized to test lung function [23]. After correct connection of the animal, pneumatic sensor and pressure sensor, a calm breath was recorded for a baseline value. Then, the parameters of lung volume, static and dynamic lung function including forced expiratory volume in 100 ms (FEV0.1), functional residual capacity (FRC), and airway resistance (RI), were measured automatically via the spirometer.

### 2.6. H&E Staining and Immunohistochemistry

After being extracted and washed in PBS, lung tissue from mice was fixed in formalin and embedded in paraffin sections via standard protocols. Hematoxylin and eosin staining was performed to assess histology. The mean linear intercept (Lm) was calculated from the number of intercepts, the length of the line, and the number of times the line was placed on the sections [24, 25]. Detections of Foxp3 and ROR*γ*t protein expression were shown by immunohistochemistry through specific antibodies binding. Anti-Foxp3 and anti-ROR*γ*t antibodies were purchased from Abcam (United Kingdom). Motic Images Advanced 3.2 was applied to analyze the intensity of protein expression.

### 2.7. Western Blot

Lung tissue from mice was ground into homogenates at the condition of 4°C for protein detection [26]. Total protein was extracted via a Total Protein Extraction Kit (Invent Biotechnologies Inc., USA) according to the manufacturer's protocol. Enhanced BCA Protein Assay Kit (Beyotime, Jiangsu, China) was used for concentration detection. Protein samples were then separated by SDS-PAGE gel electrophoresis on 12% glycine-based gels and transferred to nitrocellulose membranes (Millipore Corp., Billerica, MA, USA). Membranes were blocked in 5% (*w/v*) skimmed milk for 2 h and incubated with antibodies in 5% (*w/v*) BSA overnight at 4°C. Anti-Foxp3 antibody, anti-ROR*γ*t antibody, and anti-GAPDH antibody were purchased from Abcam (United Kingdom). Membranes were then incubated with secondary HRP-labeled antibody (Sigma-Aldrich, St. Louis, MO, USA) in 5% (*w/v*) BSA for 1 h. Afterwards, enhanced chemiluminescence (Beyotime, Jiangsu, China) was developed on membranes. The intensity of bands was quantified by densitometry using ImageJ software (National Institutes of Health, Bethesda, MD, USA).

### 2.8. ELISA

Blood was obtained and centrifuged at a speed of 3000 rpm for 10 min at 4°C. Serum was collected for assessing the levels of inflammatory cytokines TGF-*β* and IL-17 by Mouse ELISA Kit (RayBiotech, USA). IL-1*β*, IL-6, and TNF-*α* were quantified in lung homogenates using commercial ELISA kits (eBiosciences).

### 2.9. RNA Isolation and RT-qPCR

The lungs were collected under aseptic conditions and ground into homogenates at the condition of 4°C. After being isolated from a RNeasy mini Kit (Qiagen, USA), total RNA was reverse transcribed into cDNA using PrimeScript TM RT Reagent (Takara, Japan). Then, qPCR was presented with Roche FastStart Universal SYBR Green ROX (Roche, Sweden) to determine mRNA expression of Foxp3, ROR*γ*t, and *β*-actin (house-keeping gene). All primers were designed and purchased from Kumei (Jilin, China). The ΔΔCt method was performed to show the relative expression level of Foxp3 and ROR*γ*t mRNA. Primer sequences of mouse Foxp3, ROR*γ*t, and *β*-actin are shown as follows: mouse Foxp3 forward primer, 5′-ATCCTACCCACTGCTGGCAAAT-3′; mouse Foxp3 reverse primer, 5′-AGAGACTGCACCACTTCTCTCT-3′; mouse ROR*γ*t forward primer, 5′-GACGGCCAACTTACTCTTGGA-3′; mouse ROR*γ*t reverse primer, 5′-CTCGGAAGGACTTGCAGACAT-3′; mouse *β*-actin forward primer, 5′-GATCAAGATCATTGCTCCTCCTG-3′; and mouse *β*-actin reverse primer, 5′-AGGGTGTAAAACGCAGCTCA-3′.

### 2.10. Statistical Analysis

GraphPad Prism 6.0 was used for statistical analysis. The results are expressed as mean ± SD. One-way ANOVA-LSD was used to compare among groups. *p* value < 0.05 was considered statistically significant, and data was tested at level *α* = 0.05.

## 3. Results

### 3.1. Cigarette Smoke Caused a Weight Loss in Mice

**Figure 1:**
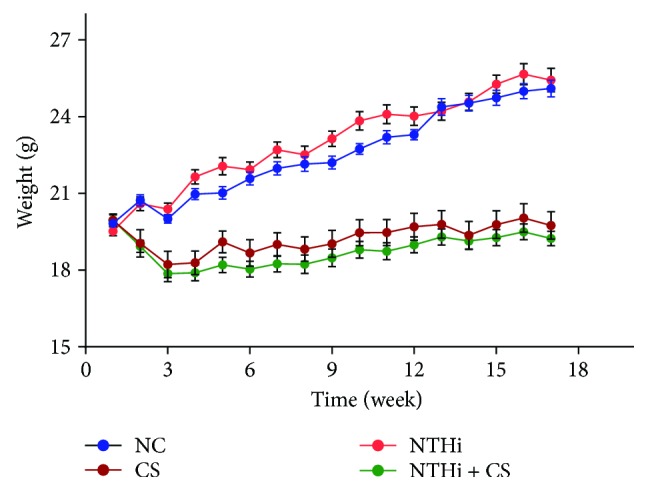
Weight change on mice during a 17-week experiment. Body weight was measured once a week, fasting 12 h before each weighing. *n* = 15 in each group. Data was expressed as mean ± SD.

We first examined a direct influence of CS and NTHi on mouse body weight. Mice were weighed once a week. As shown in Figure 1, mice in the CS and CS + NTHi groups had a significant reduced weight compared with the NC. Since mice were sacrificed 24 h after challenged with NTHi, there was no significant change between the NTHi and NC groups. These helped us to infer that CS might cause a weight loss in mice.

### 3.2. Cigarette Smoke with NTH Impaired Lung Function in Mice

**Figure 2:**
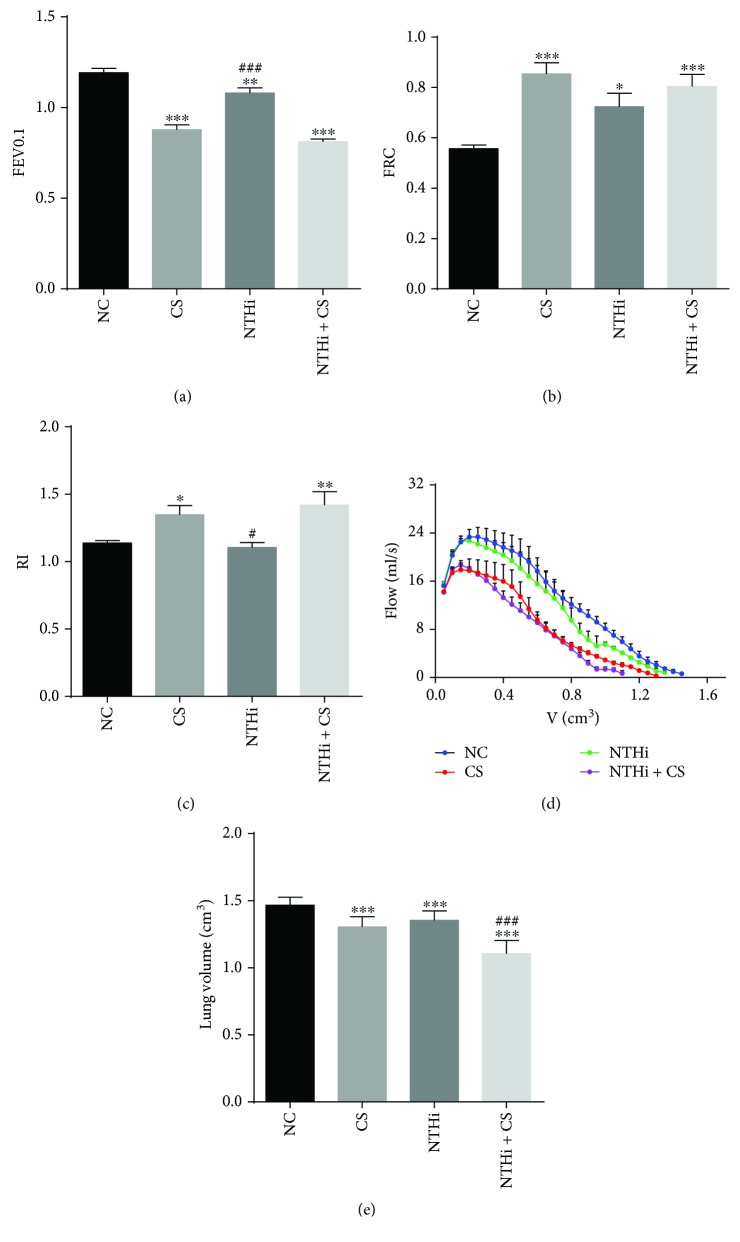
Lung function testing on mice. Invasive lung function was investigated with anesthesia and tracheal instrumentation. FEV0.1 (forced expiratory volume in 100 ms), FRC (functional residual capacity), and RI (airway resistance) in the NC, CS, NTHi, and NTHi + CS groups were shown in (a), (b), and (c), respectively. (d) Dynamic lung function, with *x*-coordinate representing lung volume and *y*-coordinate representing airflow. (e) Lung volume at the end of each inspiration. *n* = 5–7 in each group; each test was repeated three times. Data was expressed as mean ± SD. Analysis of differences among groups was conducted with one-way ANOVA-LSD. ^∗^*p* < 0.05, ^∗∗^*p* < 0.01, and ^∗∗∗^*p* < 0.001 versus NC; ^#^*p* < 0.05 and ^###^*p* < 0.001 versus CS.

Spirometry was utilized to assess lung function in this murine COPD model. Indexes as FEV0.1, FRC, and RI showed great changes in experimental groups in Figure 2. CS decreased FEV0.1 significantly (*p* < 0.001), while increased FRC (*p* < 0.001) and RI (*p* < 0.05) compared to NC. Consistent with these observations, the results of dynamic lung function indicated a decrease of lung function in all experimental groups especially in the NTHi + CS group, with smaller lung volume and lower airflow compared to NC notably at the end of each inspiration (*p* < 0.001) (Figures 2(d) and 2(e)). These results demonstrated a significant impact of CS and NTHi on lung function.

### 3.3. Morphology Impairment on Lung Tissue in the CS and NTHi Groups

**Figure 3:**
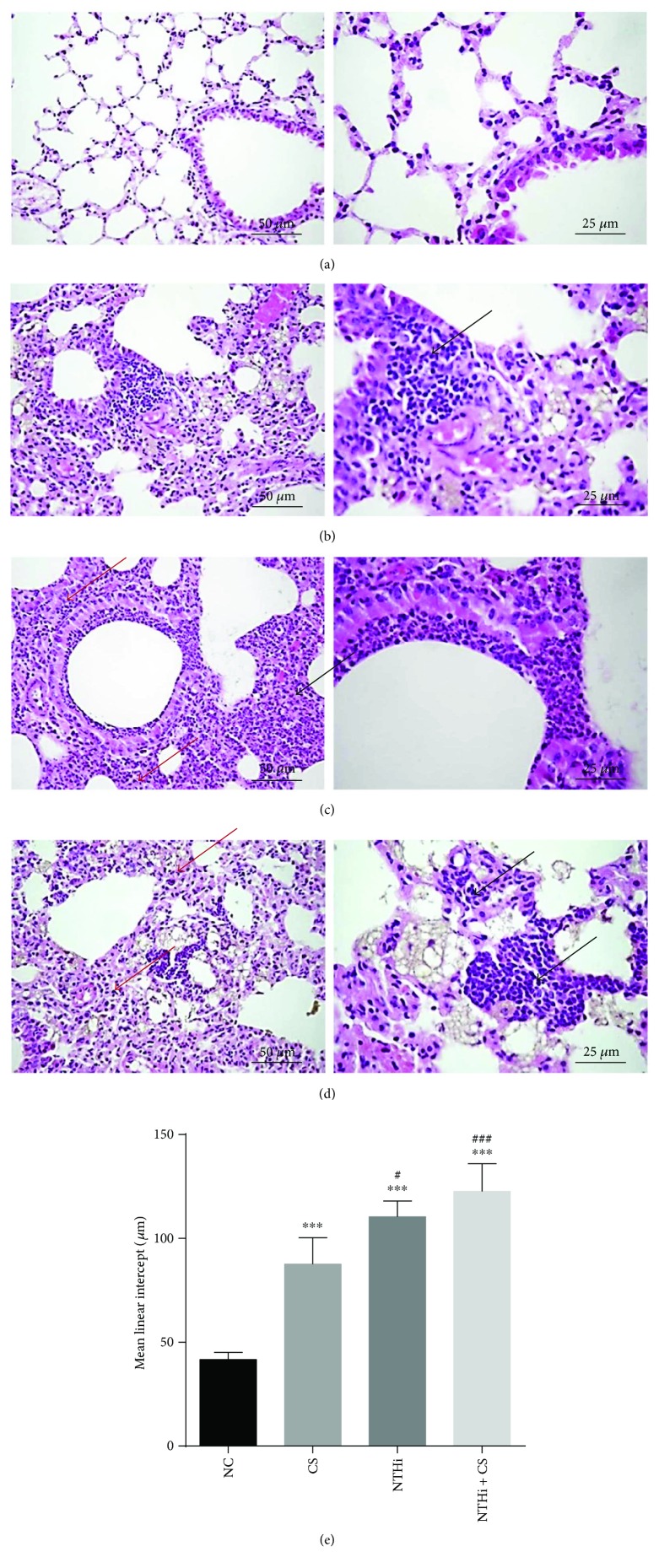
H&E staining of lung tissue in mice. (a), (b), (c), and (d) represent lung tissue sections from the NC, CS, NTHi, and NTHi + CS groups, respectively (left: 200×; right: 400×). Black arrows in (b), (c), and (d) represented the infiltrating inflammatory cells. Red arrows represented thicker alveolar septum. (e) The mean linear intercept. Lm was calculated from the number of intercepts, the length of the line, and the number of times the line was placed on the sections. *n* = 3-4 in each group. Data was expressed as mean ± SD. Analysis of differences among groups was conducted with one-way ANOVA-LSD. ^∗∗∗^*p* < 0.001 versus NC; ^#^*p* < 0.05 and ^###^*p* < 0.001 versus CS.

Changes in pulmonary morphology, integrity of epithelial cells, and infiltration of inflammatory cells were shown in Figure 3. In the NC group, there was no evident pathological change and no appreciable inflammatory cell infiltration, with integrated bronchial epithelial tissue structure (Figure 3(a)). Lung and bronchial epithelial tissue in smoke-exposed mice showed mild loss and drop. An inflammatory cell infiltration was observed in alveolar space (Figure 3(b)). Lung tissue sections presented granulocyte infiltration, enlarged airspaces, and thicker alveolar septum in the NTHi group (Figure 3(c)). The pathological damage in the NTHi + CS group was even severer, with greater expansion of the alveolar spaces. A large amount of accumulated inflammatory cells could be observed, as shown in Figure 3(d). These results revealed that CS and NTHi could both cause inflammatory and histological change in respiratory to some extent, and a combination of both agents could cause a severer damage. The mean linear intercept (Lm) was significantly higher by CS exposure (*p* < 0.001) and NTHi administration (*p* < 0.001) compared to NC, especially in the CS + NTHi group (*p* < 0.001).

### 3.4. Cigarette Smoke Together with NTHi Induced High Levels of Inflammatory Cytokines in the Lung

**Figure 4:**
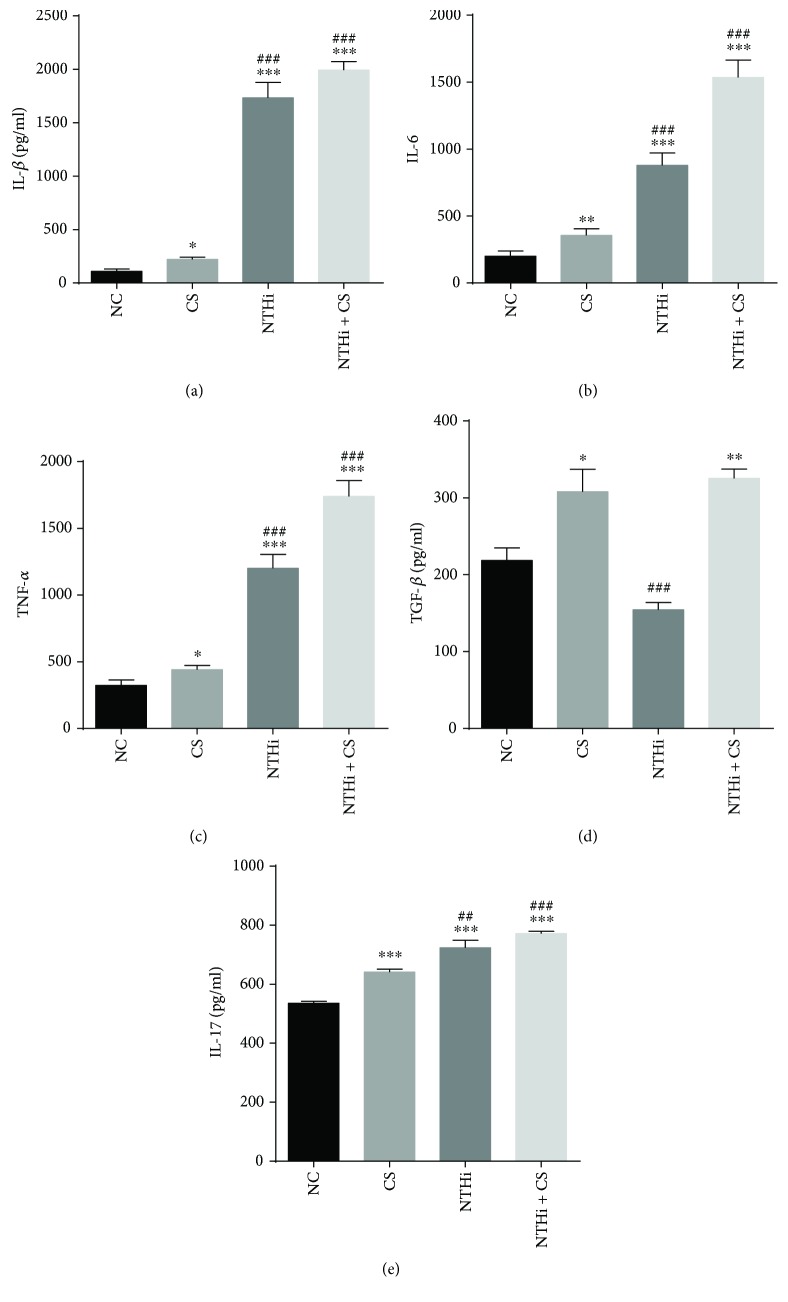
Expressions of inflammatory cytokines. IL-1*β*, IL-6, and TNF-*α* were detected in lung homogenates (a, b, and c). Serum levels of TGF-*β*1 and IL-17 in the four groups were presented in (d, e). *n* = 5–7 in each group; each test was repeated three times. Data was expressed as mean ± SD. Analysis of differences among groups was conducted with one-way ANOVA-LSD. ^∗^*p* < 0.05, ^∗∗^*p* < 0.01, and ^∗∗∗^*p* < 0.001 versus NC; ^##^*p* < 0.01 and ^###^*p* < 0.001 versus CS.

To assess the inflammatory responses in the CS and NTHi groups, ELISA was used to detect inflammatory cytokines IL-1*β*, IL-6, and TNF-*α* in lung homogenates as shown in Figures 4(a), 4(b), and 4(c). Besides, TGF-*β*1 and IL-17 were detected in serum. Compared to the NC group, mice in experimental groups showed increased inflammatory cytokine expression in the lung, especially when challenged with NTHi (*p* < 0.001). The circulating TGF-*β*1 and IL-17 (Figures 4(d) and 4(e)) in serum were presented at significantly higher concentration in the CS and NTHi + CS groups than in the NC (*p* < 0.05 and *p* < 0.01). However, there was an opposite trend in the NTHi group, where an increase of IL-17 level (*p* < 0.001) and decrease of TGF-*β*1 level (*p* < 0.001) were observed compared to the NC. These results helped to suggest an aggravating inflammation when NTHi and CS were combined and an existent role of Th17 and Tregs in this progress via the expression of TGF-*β*1 and IL-17.

### 3.5. Effects of Cigarette Smoke and NTHi on Foxp3 and ROR*γ*t Protein Expression in the Lung

**Figure 5:**
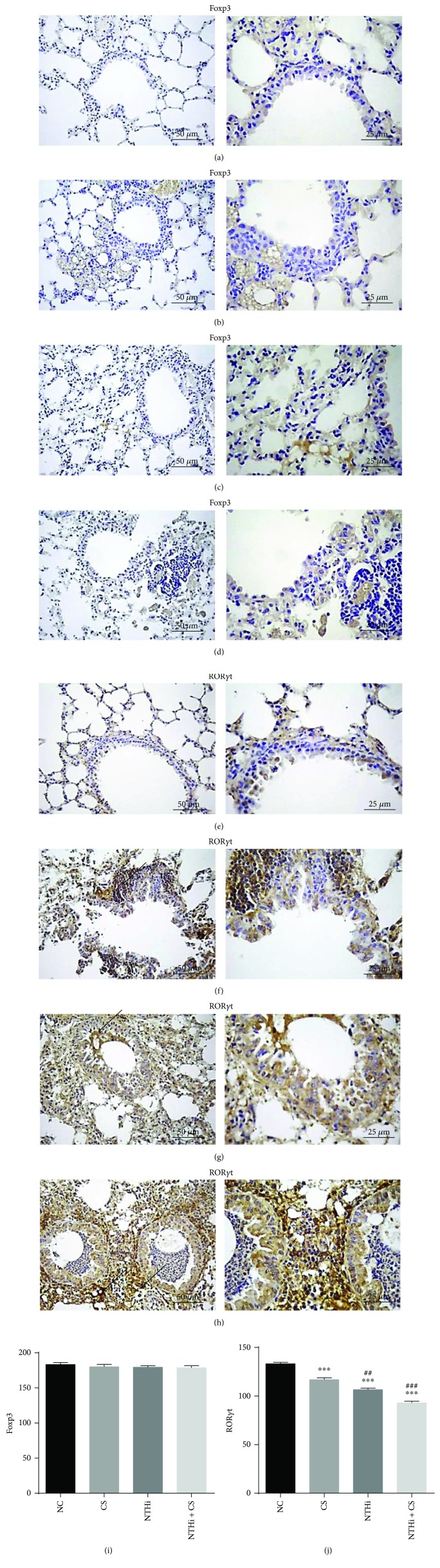
Immunohistochemical detection of Foxp3 and ROR*γ*t. Lung tissue sections were immunostained with antibodies to Foxp3 and ROR*γ*t. Color shade in (a–h) indicated the intensity of protein expression. Black arrows in (f, g, and h) represented high level of ROR*γ*t expression. Mean grey level of each section was analyzed via Motic Images Advanced 3.2, shown in (i and j). Higher grey level value meant lower positive expression. *n* = 4 in each group. Data was expressed as mean ± SD. Analysis of differences among groups was conducted with one-way ANOVA-LSD. ^∗∗∗^*p* < 0.001 versus NC; ^##^*p* < 0.01 and ^###^*p* < 0.001 versus CS.

To partially study the expressions of Tregs and Th17 cells, Foxp3 and ROR*γ*t, as biomarkers of these two subsets of CD4^+^ T cells, were detected at protein level, respectively. Immunohistochemical on lung tissue sections in Figure 5 demonstrated a similar expression of Foxp3 in each group. Compared to the NC group, the expression of ROR*γ*t increased significantly in experimental groups (*p* < 0.001, *p* < 0.001, and *p* < 0.001), especially in smoke-exposed mice when challenged with NTHi.

**Figure 6:**
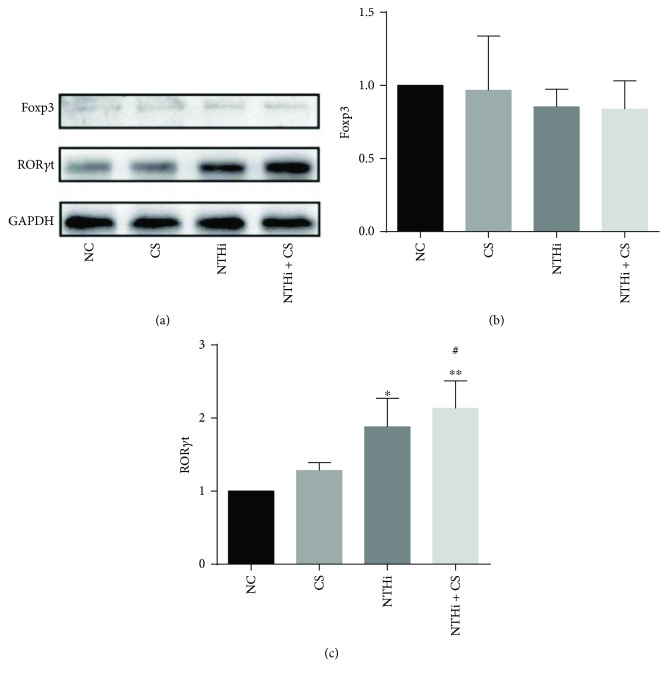
Foxp3 and ROR*γ*t expressions in lung tissue via Western blot. Band intensity was quantified by densitometry using ImageJ software (National Institutes of Health, Bethesda, MD, USA). *n* = 3 in each group. Data was expressed as mean ± SD. Analysis of differences among groups was conducted with one-way ANOVA-LSD. ^∗^*p* < 0.05 and ^∗∗^*p* < 0.01 versus NC; ^#^*p* < 0.05 versus CS.

Western blot analysis was further investigated on protein expression of Foxp3 and ROR*γ*t (Figure 6). In agreement with immunohistochemistry results, the level of Foxp3 was low without significant differences between each group. While ROR*γ*t showed a great increase in the CS and NTHi groups (*p* < 0.05), with the highest expression in the NTHi + CS group (*p* < 0.01). These results helped us to infer that CS together with NTHi could stimulate the expression of ROR*γ*t significantly in the lung at protein level. Similar expression levels of Foxp3 across the experimental groups suggested that little concentration of this protein was detected in our study, which was confirmed by quantitative histologic analysis.

### 3.6. Effects of Cigarette Smoke and NTHi on Foxp3 and ROR*γ*t at Gene Level in the Lung

**Figure 7:**
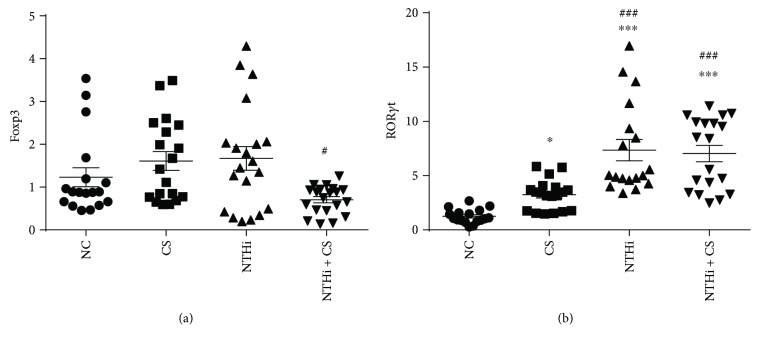
Gene expressions of Foxp3 and ROR*γ*t in lung tissue. Relative mRNA expressions were assessed by qPCR and analyzed using 2^−ΔΔCt^. *n* = 6–8 in each group; each test was repeated three times. Data was expressed as mean ± SD. Analysis of differences among groups was conducted with one-way ANOVA-LSD. ^∗^*p* < 0.05 and ^∗∗∗^*p* < 0.001 versus NC; ^#^*p* < 0.05 and ^###^*p* < 0.001 versus CS.

Relative expression of Foxp3 and ROR*γ*t at mRNA level was assessed via real-time PCR and reported as 2^−ΔΔCt^ manner (Figure 7). Compared to NC, mRNA expression of Foxp3 in the lung increased in the CS and NTHi groups but decreased in the NTHi + CS group (*p* < 0.05). ROR*γ*t expression increased significantly in experimental groups, (*p* < 0.05, *p* < 0.001, and *p* < 0.001), with the largest increase in the NTHi + CS group. Based on these results, we could infer a strong effect of Th17 cells in the lung and an attenuated function of Tregs when CS and NTHi were combined, due to the decreased expression of Foxp3.

## 4. Discussion

In this study, we have established a successful moderate COPD murine model with cigarette smoke followed by NTHi administration to investigate the inflammatory responses on the pro-/anti-inflammatory balance in COPD, reflected by Th17/Treg cell responses. CS together with NTHi worsened lung functions and inflammatory responses in mice. As a single factor, NTHi did not have too much impact on lung function, but a relative decrease of FEV0.1 and a significant increase of RI could be observed after smoking exposure and NTHi administration. It was more accurate to reflect the various airflow as lung volume changed in a dynamic lung function curve, indicating an aggravated lung function when NTHi and CS were combined, with smaller lung volume and lower airflow. The impaired lung function could probably be explained through the pathological change. The increase in FRC suggested a dynamic pulmonary hyperinflation occurring after CS stimulation, while the increase in resistance might be due to the damage of respiratory tissue, as other researchers have demonstrated [27, 28]. The thicker alveolar septae and larger expanded alveolar spaces revealed in histopathology not just weakened the lung elastic but indicated an inflammation caused by CS and NTHi, which are consistent with clinical COPD pathological features. Therefore, our model partially imitated the histopathology and lung function changes existed in COPD.

Microbiome has been widely studied and is believed to be a vital exacerbation risk in COPD patients [29]. In this study, based on the discoveries that NTHi was the most frequently isolated agent in COPD patient [7] and an important cause of exacerbations of COPD [30], we superimposed this bacterial exposure in a murine COPD model induced by CS to investigate the behavior of Tregs in inflammatory and immune responses. Consistent with our results, Ganesan et al. [31] also reported the role of NTHi in the progression of COPD. Increased goblet cells and mucin gene expression occurred during the development of lung inflammation. However, this did not happen with all pathogens, since some bacteria could attenuate the immune pathological injury in lung inflammation. Inactivated *Klebsiella* has been recently studied in both cigarette smoke-induced lung inflammation and allergic airway disease. Reductions in BAL inflammatory cytokines and systemic immune activation happened after inactivated *Klebsiella* administration, indicating a novel strategy to alter COPD pathological process and treat allergic airway disease [32, 33]. *Streptococcus pneumoniae* infection could also suppress allergic response in allergic airway disease by inducing Tregs [34, 35]. *Lactobacillus rahmnosus* and *Bifidobacterium breve* were believed to have anti-inflammatory effects in CS-induced COPD [36]. This dialectical role of pathogen infections on inflammation provided us a new field for further investigation on the impact of bacteria in respiratory disease.

Adaptive immune responses mediated by T cells play a critical role in the chronic persistent inflammation in COPD. Tregs and Th17 cells are two crucial subsets of CD4^+^ T cells that regulate inflammatory responses. Tregs have an active role in inhibiting immune responses and attenuating pulmonary inflammation [37, 38], while Th17 cells produce proinflammatory cytokines, mediating host defensive mechanisms against infection [39, 40]. TGF-*β* has been considered as an essential regulator in the generation and differentiation of Tregs converted from peripheral naive CD4^+^ T cells [41], also being protective in COPD. Zhang et al. [42] utilized a competitive receptor antagonist for TGF-*β* type I receptors to indicate that impaired TGF-*β* signaling might generate an imbalance of Th17/Treg ratio in the peripheral blood of smokers, suggesting a potential factor for COPD development. IL-17, as an important mediator in promoting tissue inflammation, could upregulate proinflammatory cytokines and chemokines [43]. Our results have shown a higher level of Foxp3 and TGF-*β*1 in the CS-induced COPD model, suggesting an increase of Tregs with a function of attenuating inflammation. However, aggravated inflammatory responses in the lung indicated that the immunosuppressive effect of Tregs was not capable of inflammatory inhibition. Consistent with our results, a clinical study on the Treg/IL-17 ratio in COPD demonstrated an increase of both proinflammatory and anti-inflammatory responses in patients with COPD, dominated by proinflammatory responses, indicating an insufficient of Tregs [44].

In this work, according to the varied expression of Foxp3 and TGF-*β*1 in the CS-NTHi stimulated murine model, we firstly found a downregulatory effect of NTHi on Tregs. TGF-*β*1, released from Tregs, dendritic cells, and macrophages, is traditionally thought to be an anti-inflammatory mediator. A dual function of TGF-*β*1 has been discovered, based on the existence of IL-6. When IL-6 is absent, TGF-*β*1 is capable of inducing Treg cell development, while in the presence of IL-6, TGF-*β*1 induces Th17 cells [12, 45, 46]. In our current study, observations of depressed Foxp3 mRNA expression and enhanced TGF-*β*1 level in the CS + NTHi group could be probably due to the increase of IL-6, switching Tregs into a Th17 response in the presence of chronic infection. However, this hypothesis needs our further investigation. Overall, Tregs were generally differentiated for an attenuating inflammatory effect via TGF-*β*1 in stable COPD against the Th17 proinflammatory effect, keeping an inflammation balance. However, after NTHi infection, Tregs were suppressed and the balance became biased, leading to the development into AECOPD.

## 5. Conclusions

In summary, our study has successfully established a COPD murine model that showed features similar to clinical COPD. NTHi infection could induce AECOPD with lower lung function and more severe inflammation. Under the condition of cigarette smoke, we inferred an attenuating inflammatory effect of Tregs. When challenged with NTHi, an impairment of Treg function was found, however, accompanied with an enhancement of Th17 inflammatory responses. Our results supported the presence of a pro-/anti-inflammatory balance in COPD reflected by Th17/Treg cell responses. NHTi infection impaired the anti-inflammatory Treg balance, and an AECOPD developed.
